# Personal Nomenclature

**Published:** 1888-02

**Authors:** J. F. Baldwin

**Affiliations:** Columbus, O.


### Correspondence.

### *PERSONAL NOMENCLATURE.*

*Editor Buffalo Medical and Surgical Journal:*

In your editorial, in the January issue of your excellent journal, you have, it seems to me, fallen into confusion—a confusion shared by nine out of every ten writers on these subjects—as to what is really meant by the "Battey operation," the "Hegar-Tait operation," and the "Tait operation." Whether or not personal nomenclature is desirable, is not the question; but if such nomenclature is to be used at all, it should be used correctly.

In the article on "Nomenclature Personal," in *Wood's Reference Handbook of the Medical Sciences*, occur definitions of these several operations. As the writer of that article, I may say that these definitions cost me more correspondence than all the other five hundred put together. The definitions of Battey's and Tait's operations are quoted verbatim from letters received from each operator.

"*Battey's Operation*—'The complete extirpation of both ovaries, while yet in a state of functional activity, for the effectual remedy of cases of disease otherwise incurable.'

"*Hegar's Operation*—The same as Battey's operation.

"*Hegar-Tait Operation*—A misnomer of Tait's operation.

"*Tait's Operation*—'Removal of the uterine appendages for physical disease other than cystoma; as the removal of the ovaries and tubes for uterine myoma; the removal of a tube for pyosalpinx, or other disease; or the removal of both ovaries and tubes for chronic inflammatory disease and adhesions.'"

The object of Battey's operation is to bring about the menopause, and thus cure diseases that would be cured by the natural arrival of that period, and, hence, it has been resorted to many times for the cure of various hysterical affections. Tait distinctly disapproves of the operation, as employed for this purpose: there must be *actual physical disease.*

Very respectfully, J. F. BALDWIN, M. D.

COLUMBUS, O., January 18, 1888.

[COMMENT.—We see no occasion to dread the charge of confusion—there has been "confusion worse confounded" about many questions in medicine, until the truth was finally evolved, it may be. All we were endeavoring to show in the portion of our editorial referred to was, that the operations advocated by various operators, and which had taken their names, began with Battey's operation; that this had been left by itself in the march of events; and that it was better to drop all individual nomenclature, which, in fact, is practically being done. We did not intend to go into the pathology of the various diseases for which these operations have been proposed, nor to discuss any technical question in detail. Perhaps, at a future time, we may treat these subjects in that manner.]

